# Review of *Dicrotendipes* Kieffer from China (Diptera, Chironomidae)

**DOI:** 10.3897/zookeys.183.2834

**Published:** 2012-04-19

**Authors:** Xin Qi, Xiao-Long Lin, Xin-Hua Wang

**Affiliations:** 1 College of Life Science, Taizhou University, Linhai, Zhejiang 317000, China; 2 College of Life Science, Nankai University, Tianjin 300071, China

**Keywords:** Chironomidae, *Dicrotendipes*, new species, key, China

## Abstract

The genus *Dicrotendipes* Kieffer from China, including 8 species, is reviewed. Two new species, *Dicrotendipes nudus* **sp. n.** and *Dicrotendipes saetanumerosus* **sp. n.** are described and the male imagines are illustrated; the record of *Dicrotendipes fusconotatus* (Kieffer) is the first for China. A key to the males of *Dicrotendipes* in China is given.

## Introduction

The genus *Dicrotendipes* was erected by Kieffer in 1913, with *Dicrotendipes septemmaculatus* (Becker, 1908) as type species. Adults of *Dicrotendipes* have been considered as pests due to large emergences (Frommer and Rauch 1971; Epler 1988), and have been implicated in allergic reactions in humans in Africa (Cranston et al. 1983). The immature stages are found in both lentic and lotic habitats, but are generally more prevalent in lentic situation. So far, there are 102 species recorded around the word.

In this paper, the Chinese material of *Dicrotendipes* is reviewed. Two new species are described, and a key to the Chinese species of *Dicrotendipes* is presented.

## Materials and methods

The morphological nomenclature follows Saether (1980). The material examined was mounted on slides, following the procedure outlined by Saether (1969). Measurements are given as ranges followed by the mean, when three or more specimens are measured, followed by the number of specimens measured (n) in parentheses. Specimens are deposited in the College of Life Science, Nankai University, China and College of Life Science, Taizhou University, China.

Abbreviations of parts measured are as follows:

**TL **Total length, Length of abdomen + length of thorax; Abdomen is measured from the concave anteriomedian margin of segment I to the apex of the gonostylus; the thorax is measured from the posterior margin of the postnotum to the anterior apex of the scutum in lateral view.

**WL **Wing length,measured from arculus to apex of wing.

**Pfe **Length of profemur.

**AR **Antennal ration,length of 11^th^ / length of flagellomeres 1–10.

**L: 5^th^/3 ^rd^**Length of the 5^th^ Palpomere / length of the 3 ^rd^ Palpomere.

**Ftu **Length of frontal tubercle.

**VR **Venarum ration, length of Cubitus (Cu) / length of Media (M).

**BV **Length of (femur + tibia + ta_1_) / length of (ta_2_ + ta_3_ + ta_4_ + ta_5_)

**LR **Leg ration, length of ta1 / length of tibia.

**SV **Length of (femur + tibia) / length of ta_1_.

**HR **Hypopygium ration,length of gonocoxite / length of gonostylus.

**HV **Hypopygium value, total length / length of gonostylus times ten.

**P_1_**Fore leg.

**P_2_** Mid leg.

**P_3_** Hind leg.

**fe **femur.

**ti** tibia.

**ta_1_…ta_n_**tarsus_1_…tarsus_n_.

**B **Brachiolum.

**R **Radius.

**R_1_** Radius 1 vein.

**R_4+5_** Radius 4+5 vein.

## Taxonomy

### Dicrotendipes flexus

(Johannsen, 1932)

http://species-id.net/wiki/Dicrotendipes_flexus

Chironomus (Limnochironomus) flexus Johannsen, 1932: 530.
Limnochironomus flexus : Lenz 1937: 6.
Dicrotendipes flexus : Hashimotoet al. 1981: 14; Epler 1988: 128; Wanget al. 1990: 29; Wang 2000: 643.

#### Specimens examined.

China, Hubei: 5♂♂, Wuhan City, Donghu Lake, 30°35.06’N, 114°22.42’E, 22.iv.1977, Wang SD sweeping method; Guangdong: 2♂♂, Fengkai County, Heishiding Nature Conservation Area, 23°29.14’N, 111°50.54’E, 18.iv.1988, Wang XH, light trap; Shandong: 1 ♂, Yantai City, Kunyu Mountain, 37°23.53’N, 121°36.42’E, 24.viii.1987, Wang XH, sweeping method.

#### Remarks.

*Dicrotendipes flexus* (Johannsen) closely resembles *Dicrotendipes nervosus* (Staeger) in the structure of hypopygium, but can be separated by the apparently disjunct distributions and fewer setae on R and R_1_, (21−26 in *Dicrotendipes flexus*, more than 35 in *Dicrotendipes nervosus*). All examined Chinese specimens comply with the description of Johannsen (1932) and Hashimoto et al. (1981).

#### Distribution.

China (Hubei, Guangdong and Shandong Province); Australia; Japan; Indonesia.

### Dicrotendipes fusconotatus

(Kieffer, 1922)

http://species-id.net/wiki/Dicrotendipes_fusconotatus

Calochironomus fusconotatus Kieffer, 1922: 68.
Calochironomus grisseonotatus Kieffer, 1922: 69.
Dicrotendipes forkficula Kieffer, 1925: 298.
Dicrotendipes nilicola Kieffer, 1925: 300.
Chironomus (Dicrotendipes) fusconotatus : Freeman 1957: 362.
Dicrotendipes fusconotatus : Contreras-Lichtenberg 1986: 717.

#### Specimens examined.

China, Jiangxi: 5♂♂, Yongxiu County, Nanji Town, 28°56.42’N, 116°21.37’E, 12.vi.2004, Yan CC, light trap.

#### Remarks.

Chinese specimens mainly agree with the description of Freeman (1957), but vary in the coloration of the abdomen: the abdomen of the Chinese species is black; while in Freeman (1957), the abdomen is light green and the median of each abdominal tergite black.

#### Distribution.

China (Jiangxi Province); Belgium; Congo; Egypt; Israel; Kenya; Sudan; Zaire.

### Dicrotendipes nervosus

(Staeger, 1839)

http://species-id.net/wiki/Dicrotendipes_nervosus

Chironomus nervosus Staeger, 1839: 567.
Tendipes (Dicrotendipes) nervosus : Dendy and Sublette 1959: 514.
Chironomus (Dicrotendipes) nervosus : Sublette 1964: 126.
Dicrotendipes nervosus : Epler 1988: 63; Wanget al. 1990: 29; Wang 2000: 643.

#### Specimens examined.

China, Jiangxi: 7♂♂, Yongxiu County, Nanji Town, 28°56.42’N, 116°21.37’E, 12.vi.2004, Yan CC, light trap; Ningxia: 3♂♂, Yinchuan City, 38°29.23’N, 106°13.19’E, Wang XH, light trap; Shandong: 2♂♂, Zaozhuang City, Baodugu Mountain, 34°59.11’N, 117°43.07’E, 28.v.1994, Wei MC, sweeping method; Tianjin: 6♂♂, Yuqiao Reservoir, 40°02.35’N, 117°27.01’E, 17.x.1987, Wang XH, light trap; Zhejiang: 1♂, Quzhou City, Yunxi village, 29°01.15’N, 118°56.51’E, 20.iv.2011, Lin XL, sweeping method.

#### Distribution.

China (Jiangxi, Shandong, Zhejiang Province, Ningxia Hui Autonomous Region and Tianjin City); Brazil; Britain; Canada; Denmark; Germany; Japan; Netherlands; Korea; Sweden; Russia; USA.

### Dicrotendipes nudus sp. n.

urn:lsid:zoobank.org:act:C0444659-611D-4DAB-BDD3-1AD246E9978F

http://species-id.net/wiki/Dicrotendipes_nudus

Figs 1−5

#### Diagnosis.

R_1_ and R_4+5_ without seta; tergite IX without median seta; anal point with basal peduncle and bulbous ventral extension, 6−9 dorsal basal setae and 6 lateral setae.

#### Description.

Male imago (n = 18)

TL 2.65−3.20, 2.95 mm. WL 1.65−2.00, 1.82 mm. TL/WL 1.58−1.94, 1.72. WL/Pfe 2.14−2.43, 2.31.

Coloration.Head, thorax and abdominal tergite VI−IX brown, abdominal tergite I−V pale yellow; legs yellowish-brown.

Head. AR 1.85−2.12, 2.02. Temporal setae 10−16, 13. Clypeus with 12−19, 16 setae. Tentorium 100−163, 146 µm long, 20−35, 28 µm wide. Palpomere lengths (in µm): 34−42, 35; 43−55, 48; 40-45; 108−130, 121; 130−148, 138; 163−215, 179. L: 5^th^/3 ^rd^ 1.35−1.67, 1.58. Frontal tubercle 10.20−17.50, 14.20 µm long, 5.00−7.50, 6.20 µm wide.

**Figures 1–5.**
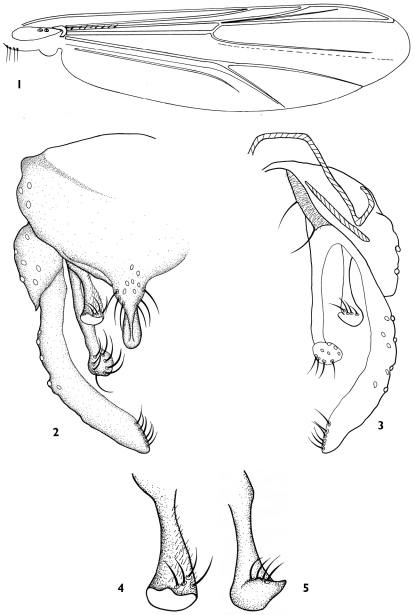
*Dicrotendipes nudus* sp. n., male **1** wing **2** hypopygium (dorsal view ) **3** hypopygium (ventral view ) **4**–**5** superior volsella.

Wing (Fig. 1).Wing transparent, without markings. VR 1.11−1.16, 1.13. B 1−3, 2 setae; R with 7−11, 9 setae; R_1_ and R_4+5_ without seta. Squama with 4−6, 5 setae.

Thorax.Dorsocentrals 8−11, 10; acrostichals 4−5, 4; prealars 3−4, 4. Scutellum with 4−9, 7 setae.

Legs. Fore tibia with rounded scale lacking spur. Spurs on mid tibiae 23–25, 24 µm and 18−25,20 µm long, including combs 26–32, 30 µm and 26–32, 30 µm long; spurs on hind tibia 22–32, 26 µm and 18−20,19 µm long including combs 24–28, 26 µm and 22–25, 23 µm long. Width at apex of front tibia 53−58, 55 µm, of mid tibia 50−55, 53 µm, of hind tibia 55−65, 59 µm. Lengths (in µm) and proportions of legs in Table 1.

Hypopygium(Figs 2−5). Anal point 40−60, 50 µm long, with basal peduncle and bulbous ventral extension, 6−9 dorsal basal setae and 6 lateral setae. Tergite IX without median setae; laterosternite IX with 3−4, 3 setae. Phallapodeme 95−103, 97 µm long; transverse sternapodeme 40−50, 45 µm long, laterally narrowed, medially broad, inverted U-shaped. Gonocoxite 142−165, 156 µm long. Superior volsella 83−92, 85 µm long, 23−27, 25 µm wide; digitiform with short ventral extension; with numerous micro setae and 3−4 short apical setae (Figs 4−5). Inferior volsella 128−155, 142 µm long; elongate, apex bulbiform, with 6−9, 8 apical setae in 2 rows. Gonostylus 150−195, 172 µm long; slightly curved medially, with 5−7, 6 apical setae along inner margin. HR 0.73−1.17, 0.82; HV 1.82−1.88, 1.85.

#### Type materials.

Holotype: 1♂, China, Hebei: Chicheng County, 40°54.16’N, 115°54.08’E, 21.vii.2001, Guo YH, light trap. Paratypes (17): Hebei: 2♂♂, Chicheng County, 40°54.16’N, 115°54.08’E, 21.vii.2001, Guo YH, light trap; Xinjiang: 5♂♂, Hebahe County, 48°04.30’N, 86°24.47’E, 15.vii.2002, Tang HQ, light trap; Zhejiang: 3♂♂, Ningbo City, 29°48.36’N, 121°34.53’E, 10.v.2010, Qi X, sweeping method; 1♂, Sanmen County, 29°05.55’N, 121°23.45’E, 28.vii.2010, Lin XL, sweeping method; 6♂♂, Tiantai County, Huading Mountain, 29°14.51’N, 121°06.31’E,13.iv.2011, Lin XL, light trap.

#### Etymology.

The species name is from Latin, *nudus*, meaning bare, referring to R_1_ and R_4+5_ without seta, which is unique within the genus.

#### Remarks.

*Dicrotendipes nudus* closely resembles *Dicrotendipes nervosus*, but can be separated by R_1_ and R_4+5_ of *Dicrotendipes nudus* without seta; while in *Dicrotendipes nervosus*, R_1_ with 11−20, 15 setae, R_4+5_ with17−28, 22 setae.

#### Distribution.

The species is known from Hebei, Zhejiang Province and Xinjiang Uygur Autonomous Region of China.

**Table 1.** Lengths (in µm) and proportions of legs of *Dicrotendipes* nudus sp. n.

	P1	P2	P3
fe	710−850, 788	670−790, 752	770−870, 818
ti	560−640, 600	570−710, 638	800−940, 870
ta1	890−1050, 991	310−380, 338	490−560, 528
ta2	380−460, 428	180−220, 197	260−300, 278
ta3	310−380, 353	110−140, 127	210−240, 218
ta4	240−300, 280	70−80, 77	110−130, 123
ta5	130−150, 143	71−83, 77	90−100, 95
LR	1.59−1.75, 1.65	0.52−0.55, 0.53	0.59−0.64, 0.61
BV	1.89−2.04, 1.96	3.45−4.87, 3.84	3.06−3.19, 3.11
SV	1.32−1.43, 1.37	4.03−4.17, 4.11	3.08−3.29, 3.20

### Dicrotendipes pelochloris

(Kieffer, 1912)

http://species-id.net/wiki/Dicrotendipes_pelochloris

Tendipes pelochloris Kieffer, 1912: 39; Kieffer 1916: 113.
Limnochironomus niveicauda Kieffer, 1921: 585.
Chironomus (Limnochironomus) niveicauda : Johannsen 1932: 528.
Dicrotendipes niveicauda : Sublette and Sublette 1973: 404; Hashimoto et al. 1981: 13.
Chironomus inferior Johannsen, 1932: 534.
Cladotendipes inferior : Lenz 1937: 7.
Dicrotendipes inferior : Sublette and Sublette 1973 1973: 403.
Chironomus (Dicrotendipes) wirthi Freeman, 1961: 692.
Dicrotendipes pelochloris : Epler 1988: 134; Wanget al. 1990: 28; Wang 2000: 644.

#### Specimens examined.

China, Hainan: 2♂♂, Xinglong County, Huaqiao Farm, 18°43.27’N, 110°14.42’E, 21.v.1985, Wang XH, light trap; Hebei: 1♂, Qinhuangdao City, 39°55.53’N, 119°36.19’E, 4.vi.1985, Li HH, sweeping method; 3♂♂, Chicheng County, 40°54.16’N, 115°54.08’E, 21.vii.2001, Guo YH, light trap; Jiangxi: 2♂♂, Yongxiu County, Nanji Town, 28°56.42’N, 116°21.37’E, 12.vi.2004, Yan CC, light trap; Fujian: 11♂♂, Shanghang County, 25°02.32’N, 116°26.12’E, 6.v.1993, Wang XH, light trap; 2♂♂, Longyan City, 25°07.14’N, 117°02.20’E, 25.ix.2002, Liu Z, light trap; Guangxi: 4♂, Leye County, 24°47.30’N, 106°33.47’E, 24.vii.2004, Yu X, light trap; Guizhou: 2♂♂, Guiyang City, Huaxi, 26°24.32’N, 106°38.58’E, 23.vii.1995, Bu WJ, sweeping method; Taiwan: 2♂♂, Taibei City, 25°08.33’N, 121°36.57’E, 21.vii.2003, Wang XH, light trap.

#### Remarks.

The Chinese specimens mainly agree with the description by Epler (1988). According to Epler (1988), there was some variation in the coloration of the wing in *Dicrotendipes pelochloris*, from hyaline to dusky brown, or with diffuse brown cloud along R_1_, R_4+5_, M, Cu and An. The wings of Chinese specimens are hyaline, without markings. The Chinese specimens are smaller than the specimens described in Epler (1988). Some measured differences between the Chinese specimens and the specimens described by Epler (1988) are shown in Table 2.

#### Distribution.

China (Hainan, Hebei, Fujian, Guizhou, Jiangxi, Taiwan Province and Guangxi Zhuang Autonomous Region); Australia; India; Indonesia; Japan; Pakistan; Philippines; South Korea.

**Table 2.** Differences between the specimens of China and of Epler (1988)

	Chinese specimens	Description of Epler (1988)
TL	2.68−4.25, 3.55 mm	3.74−4.40, 4.01 mm
WL	1.38−2.43, 1.82 mm	1.73−2.28, 1.96 mm
Ftu	13−33, 19 µm	16−26, 20 µm
AR	1.91−2.44, 2.17	1.95−2.27, 2.09
VR	1.05−1.14, 1.10	0.81−0.92, 0.85
LR1	1.58−1.84, 1.73	1.66−2.07, 1.86
BV1	1.71−2.75, 1.87	1.78−1.98, 1.89
BV2	3.66−4.27, 3.89	4.06−4.74, 4.22
SV2	3.72−4.17, 3.97	3.98−4.38,4.17

### Dicrotendipes saetanumerosus sp. n.

urn:lsid:zoobank.org:act:B8666895-7A48-41E0-8799-8B236E7FDDAD

http://species-id.net/wiki/Dicrotendipes_saetanumerosus

Figs 6−8

#### Diagnosis.

Tergite IX with more than 30 median setae; anal point broad, bare; superior volsella pediform, with 11−16 lateral setae.

#### Description.

Male imago (n = 7)

TL 3.65−4.30, 3.82 mm. WL 1.80−2.30, 2.10 mm. TL/WL 1.87−2.03, 1.93. WL/Pfe 1.86−2.04, 1.96.

Coloration.Head, thorax and abdominal tergite VII−IX brown, abdominal tergite I−VI pale yellow; legs yellowish-brown.

Head. AR 2.38−2.55, 2.40. Temporal setae 19−22, 20. Clypeus with 16−20, 17 setae. Tentorium 120−155, 136 µm long, 26−35, 30 µm wide. Palpomere lengths (in µm): 32−53, 45; 58−68, 62; 155−185, 167; 165−195, 172; 235−260, 241. L: 5^th^/3 ^rd^ 1.41−1.52, 1.46. Frontal tubercle 7.50−15.00, 10.00 µm long, 5.00−6.50, 5.52 µm wide.

**Figures 6–8.**
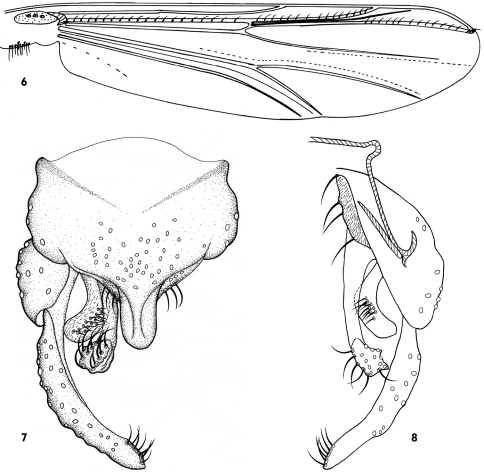
*Dicrotendipes saetanumerosus* sp. n., male **6** wing **7** hypopygium (dorsal view ) **8** hypopygium (ventral view ).

Wing (Fig. 6).Wing transparent, without markings. VR 1.05−1.06, 1.05. B 2−3, 2 setae; R with17−20, 18 setae; R_1_ with 12−16, 14 setae; R_4+5_ with 17−19, 18. Squama with 4−9, 6 setae.

Thorax.Dorsocentrals 8−11, 10; acrostichals 9−16, 12; prealars 4−5, 4. Scutellum with 8−11, 9 setae.

Legs. Fore tibia with rounded scale lacking spur. Spurs on mid tibiae 23–28, 26 µm and 25−30, 26 µm long, including combs 20–23, 21 µm and 15–18, 16 µm long; spurs on hind tibia 23−28, 26 µm and 25−30, 27 µm long including combs 20–23, 21 µm and 15–18, 16 µm long. Width at apex of front tibia 58−68, 60 µm, of mid tibia 58−73, 63 µm, of hind tibia 63−85, 70 µm. Lengths (in µm) and proportions of legs in Table 3.

Hypopygium(Figs 7−8). Anal point 40−50, 45 µm long, broad, bare. Tergite IX with more than 30 median setae; laterosternite IX with 2−4, 3 setae. Phallapodeme 90−115, 97 µm long; transverse sternapodeme 40−50, 45 µm long, laterally narrowed, medially broad, inverted U-shaped. Gonocoxite 165−230, 180 µm long. Superior volsella 68−77, 70 µm long, 38−68, 50 µm wide; pediform, with 11−16 lateral setae. Inferior volsella 138−163, 142 µm long; elongate, apex bulbiform, with 9−12, 10 apical setae in 2 rows. Gonostylus 180−195, 186 µm long; slightly curved medially, with 5−7, 6 apical setae along inner margin. HR 0.80−0.90, 0.82; HV 1.83−2.05, 1.87.

#### Type materials.

Holotype: 1♂, China, Shandong: Taian City, Tai Moutain 36°11.37’N, 117°08.13’E, 25.v.1994, Wang XH, light trap. Paratypes (8): Shandong: 1♂, Taian City, Tai Moutain, 36°11.37’N, 117°08.13’E, 25.v.1994, Wang XH, light trap; Hubei: 2♂♂, Shiyan City, Wudang Mountain, 32°30.22’N, 111°05.09’E, 16.vii.1997, Wang BX, light trap; Zhejiang: 5♂♂, Kaihua County, 29°05.57’N, 118°23.19’E, 13.iv.2011, Lin XL, light trap.

#### Etymology.

The species name is from Latin, *saeta*, meaning setae, *numerosus*, meaning numerous, referring to the tergite IX of the species with more than 30 setae, which is unique within the genus.

#### Remarks.

*Dicrotendipes saetanumerosus* sp. n. closely resembles *Dicrotendipes tamaviridis* Sasa, 1981 in the structure of hypopygium, but the new species *Dicrotendipes saetanumerosus* can be separated from *Dicrotendipes tamaviridis* on the basis of following points: (1) the anal point of *Dicrotendipes saetanumerosus* sp. n. is broad and not expanded apically, but the anal point of *Dicrotendipes tamaviridis* is slender and expanded apically; and (2) the tergite IX in *Dicrotendipes saetanumerosus* sp. n. has more than 30 median setae, while *Dicrotendipes tamaviridis* has nomedian setae and 8−9 setae in the base of anal point.

#### Distribution.

The species is known from Hubei, Shandong and Zhejiang Province of China.

**Table 3.** Lengths (in µm) and proportions of legs of *Dicrotendipes saetanumerosus* sp. n.

	P1	P2	P3
fe	970−1125, 1010	840−990, 890	950−1125, 1000
ti	750−780, 760	710−840, 750	970−1150, 1000
ta1	1400−1600, 1500	390−470, 432	620−750, 674
ta2	650−680, 660	220−270, 240	310−390, 350
ta3	525−580, 550	150−180, 160	260−310, 280
ta4	450−500, 470	90−120, 110	150−190, 170
ta5	225−270, 240	70−90, 80	90−120, 115
LR	1.87−1.96, 1.92	0.55−0.58, 0.56	0.64−0.72, 0.66
BV	1.73−1.78, 1.76	3.48−3.66, 3.54	3.00−3.29, 3.14
SV	1.76−1.95, 1.83	2.47−2.76, 2.55	4.15−5.01, 4.26

### Dicrotendipes septemmaculatus

(Becker, 1908)

http://species-id.net/wiki/Dicrotendipes_septemmaculatus

Chironomus septemmaculatus Becker, 1908: 77.
Dicrotendipes pictipennis Kieffer, 1913: 23; Freeman 1955: 22.
Dicrotendipes formosanus Kieffer, 1916: 115; Hashimoto 1981: 12.
Dicrotendipes formosanus var *frontalis* Kieffer, 1916: 116.
Dicrotendipes frontalis : Sublette and Sublette 1973: 403.
Dicrotendipes speciosus Kieffer, 1924: 256; Kieffer 1925: 299.
Dicrotendipes quatuordecimpunctatum (Goetghebuer, 1936): Contreras-Lichtenberg 1986: 710.
Dicrotendipes septemmaculatus : Epler 1988: 42; Wanget al. 1990: 28; Harrison 1993: 363; Spies and Saether 2004: 41.

#### Specimens examined.

China, Hebei: 3♂♂, Qinhuangdao City, 39°55.53’N, 119°36.19’E, 4.vi.1985, Li HH, sweeping method; Guizhou: 2♂♂, Guiyang City, Huaxi, 26°24.32’N, 106°38.58’E, 23.vii.1995, Bu WJ, sweeping method; 1♂, Libo County, Maolan Town, 25°17.21’N, 108°04.28’E, 28.vii.1995, Bu WJ, sweeping method; Shandong: 1♂, Taian City, Tai Moutain, 36°11.37’N, 117°08.13’E, 25.v.1994, Wang XH, light trap; Taiwan: 3♂♂, Taibei City, 25°08.33’N, 121°36.57’E, 21.vii.2003, Wang XH, light trap; Yunnan: 1♂, Wuding County, Shishan Moutain, 25°31.58’N, 102°22.32’E, 8.vii.1986, Wang XH, sweeping method; 1♂, Eryuan County, 26°19.56’N, 100°02.03’E, 18.vii.1986, Wang XH, light trap; 2♂♂, Kunming City, 25°04.09’N, 102°42.14’E, Bu WJ, sweeping method; 2♂♂, Dali City, Yinqiao Town, 25°45.16’N, 100°07.31’E, 22.v.1996, Wang XH, sweeping method.

#### Remarks.

The wing spots are variable in *Dicrotendipes septemmaculatus*. They may be absent in teneral specimens, and the pair of spots in cell r_4+5_ is sometimes combined into one spot. The Chinese specimens have one spot in cell r_4+5_.

#### Distribution.

China (Hubei, Guizhou, Shandong, Taiwan and Yunnan Province); Algeria; Australia; Burma; Egypt; Bangladesh; India; Indonesia; Japan; Lebanon; Namibia; Nigeria; South Africa; Spain; Sundan; Uganda; Zimbabwe; Zaire.

### Dicrotendipes tamaviridis

Sasa, 1981

http://species-id.net/wiki/Dicrotendipes_tamaviridis

Dicrotendipes tamaviridis Sasa, 1981: 99; Niitsuma 1995: 444; Wang 2000: 644.

#### Specimens examined.

China, Hubei: 3♂♂, Shiyan City, Wudang Mountain, 32°30.22’N, 111°05.09’E, 16.vii.1997, Wang BX, light trap; Gansu: 1♂, Dingxi City, Min County, 34°26.34’N, 104°02.20’E, 16.v.1993, Yang ZC, light trap; Shaanxi: 1♂, Liuba County, 33°37.16’N, 106°55.12’E, 2.vii.1994, Bu WJ, light trap; Zhejiang: 6♂♂, Kaihua County, 29°05.57’N, 118°23.19’E, 13.iv.2011, Lin XL, light trap.

#### Remarks.

Sasa (1981) described this species based on material from Japan and Niitsuma (1995) described the pupae, larvae and adults. Chinese specimens agree with the adult description of Niitsuma (1995). Some measured differences between the Chinese specimens and the specimens described by Niitsuma (1995) are shown in Table 4.

#### Distribution.

China (Hubei, Gansu, Shaanxi and Zhejiang Province); Japan.

**Table 4.** Differences between the specimens of China and of Japan

	Chinese specimens	Japanese specimens
TL	2.94−3.60 mm	2.5−3.3 mm
Ftu	7.5−10 µm	3−10 µm
AR	1.85−2.21	1.9−2.3
VR	1.12−1.14	0.81−0.92, 0.85

##### Key to males of the genus *Dicrotendipes* in China

**Table:** 

1	R_4+5_ without setae	*Dicrotendipes nudus* sp. n.
–	R_4+5_ with setae	2
2	Small, membranous, triangular flap-like appendages present near base of anal point	*Dicrotendipes fusconotatus* (Kieffer)
–	Base of anal point without appendages	3
3	Inferior volsella deeply bifid apically	*Dicrotendipes septemmaculatus* (Becker)
–	Inferior volsella with simple apex or apex bulbiform	4
4	Tergite IX with median setae	5
–	Tergite IX without median setae	6
5	Anal point sharply reflexed ventrad; tergite IX with 6−14 setae	*Dicrotendipes pelochloris* (Kieffer)
–	Anal point not sharply reflexed ventrad; tergite IX with more than 30 setae	*Dicrotendipes saetanumerosus* sp. n.
6	Wing with more than 35 setae on R & R_1_	*Dicrotendipes nervosus* (Staeger)
–	Wing with less than 30 setae on R & R_1_	7
7	Superior volsella with 3 short setae; cylindrical, curving outward; apex bare, expanded	*Dicrotendipes flexus* (Johannsen)
–	Superior volsella with 9−10 short setae; pediform, apex not expanded	*Dicrotendipes tamaviridis* Sasa

## Supplementary Material

XML Treatment for Dicrotendipes flexus

XML Treatment for Dicrotendipes fusconotatus

XML Treatment for Dicrotendipes nervosus

XML Treatment for Dicrotendipes nudus

XML Treatment for Dicrotendipes pelochloris

XML Treatment for Dicrotendipes saetanumerosus

XML Treatment for Dicrotendipes septemmaculatus

XML Treatment for Dicrotendipes tamaviridis
